# Underestimated pyrazinamide resistance may compromise outcomes of pyrazinamide containing regimens for treatment of drug susceptible and multi-drug-resistant tuberculosis in Tanzania

**DOI:** 10.1186/s12879-019-3757-1

**Published:** 2019-02-07

**Authors:** Saumu Pazia Juma, Athanasia Maro, Suporn Pholwat, Stellah G. Mpagama, Jean Gratz, Alphonse Liyoyo, Eric R. Houpt, Gibson S. Kibiki, Blandina T. Mmbaga, Scott K. Heysell

**Affiliations:** 1 Kilimanjaro Clinical Research Institute, Moshi, Tanzania; 2 Kilimanjaro Christian Medical Centre and Kilimanjaro Christian Medical University College, Kilimanjaro, Tanzania; 3 Kibong’oto Infectious Diseases Hospital, Kilimanjaro, Tanzania; 4 Division of Infectious Diseases and International Health, University of Virginia, Charlottesville, VA USA; 5 East African Health Research Commission, Arusha, Tanzania

## Abstract

**Background:**

Tuberculosis (TB) is the leading cause of death from an infectious disease and the roll-out of rapid molecular diagnostics for rifampin resistance has resulted in a steady rise in the number of patients with multidrug-resistant (MDR)-TB referred for treatment. Pyrazinamide is used in susceptible TB treatment for 6 months when used in combination with rifampin, isoniazid and ethambutol and is an important companion drug in novel MDR-TB trials. This study was undertaken to determine the prevalence of pyrazinamide resistance by either phenotypic or *pncA* testing among patients admitted to a referral hospital in Tanzania for drug-susceptible and MDR-TB treatment.

**Methods:**

Surveillance sputa were sent among subjects beginning TB therapy at the national MDR-TB referral hospital during a 6 month period in 2013–2014. Mycobacterial cultures of pretreatment sputa were performed at the Kilimanjaro Clinical Research Institute (KCRI) in the BACTEC mycobacterial growth indicator tubes (MGIT) 960 system. Speciation of *M. tuberculosis* complex was confirmed by MTBc assay. Isolates were sub-cultured on to Lowenstein-Jensen (LJ) slants. Phenotypic resistance to pyrazinamide was performed in the MGIT system while a real-time PCR with High Resolution Melt (HRM) technique was used to determine mutation in the *pncA* gene from the same pure subculture. Sputa were then collected monthly to determine the time to culture negativity. Final treatment outcome was determined.

**Results:**

Ninety-one *M. tuberculosis* isolates from individual patients were available for analysis of which 30 (32.9%) had MDR-TB, the mean (±SD) age was 33 ± 10 years, and the majority 23 (76.7%) were males. Of the 30 MDR-TB patients, 15(50%) had isolates with pyrazinamide resistance by conventional MGIT testing. This proportion expectedly exceeded the number with pyrazinamide resistance in the 61 patients without MDR-TB, 13 (21.3%) (*p* = 0.008). Six (20%) of MDR-TB patients had a poor outcome including treatment failure. Among patients with treatment failure, 5 (83%) had pyrazinamide resistance compared to only 10 (41.6%) with treatment success (*p* = 0.08). Two patients died, and both had isolates with pyrazinamide resistance. No other pretreatment characteristic was associated with treatment outcome.

**Conclusion:**

Pyrazinamide susceptibility appears to be important in clinical outcomes for MDR-TB patients, and susceptibility testing appears to be a critical adjunct to TB care. The high proportion of PZA resistance in non-MDR TB cases calls for further local investigation.

## Background

Tuberculosis (TB) is the leading cause of death from an infectious disease and Tanzania is classified as one of the 22 high burden countries for TB [1, 2]. Multidrug-resistant (MDR)-TB, defined as resistance to the most potent first-line TB drugs, isoniazid and rifampin, considerably compromises TB treatment success due to delayed diagnosis of drug-resistance, reduced potency and increased toxicity of second-line TB drugs, and prolonged treatment courses. MDR-TB surveillance is limited in Tanzania [3], but a steady rise in the number of patients referred for MDR-TB treatment has been observed, and attributed to the roll-out of rapid molecular diagnostics for rifampin resistance [4].

In Tanzania, treatment for MDR-TB was made available since 2009 and remains coordinated at the national MDR-TB referral hospital, Kibong’oto Infectious Diseases Hospital (KIDH) [5]. KIDH also serves as a regional referral hospital for other non-MDR-TB cases. MDR-TB treatment at KIDH follows WHO guidelines and consists of a largely empiric drug regimen including pyrazinamide (PZA) which patients are prescribed without susceptibility testing. Importantly, PZA is used for susceptible TB treatment for 6 months when used in combination with rifampin, isoniazid and ethambutol [6, 7]. In studies of MDR-TB where PZA susceptibility testing was retrospectively performed, PZA resistance has been independently associated with poor outcome [8]. Furthermore, the WHO has recently endorsed a shorter course regimen for MDR-TB that includes PZA, but known resistance to any drug in the regimen is a relative contraindication to the use of the shorter course regimen [7]. PZA has also been shown to enhance the in vitro and animal model activity of the novel TB agents, including pretomanid and bedaquiline, which has prompted numerous large TB treatment trials to test novel or newly approved drugs in PZA containing regimens [7, 9–11].

PZA is a pro-drug that requires conversion into its active form, pyrazinoic acid by the bacterial enzyme pyrazinamidase, which is encoded by the gene *pncA* [9]. Susceptibility testing to PZA is cumbersome but can be performed by conventional phenotypic assays which are considered the gold standard, lower sensitivity enzymatic testing for the presence of functional PZase, or by higher-cost molecular diagnostics examining for mutations in *pncA* [10]. KIDH has an ongoing collaboration with the Kilimanjaro Clinical Research Institute (KCRI) that has proficiency in both phenotypic and molecular diagnostics for PZA susceptibility testing. Hence, a study was undertaken to determine the prevalence of PZA resistance by either phenotypic or *pncA* testing among patients admitted to KIDH for drug-susceptible and MDR-TB treatment. Among MDR-TB patients, investigation was made to determine if any pretreatment characteristic was more common among those with PZA resistance and whether PZA resistance influenced treatment outcome.

## Methods

### Study site

KIDH is forty kilometers to KCRI-Biotechnology laboratory which performs smear microscopy, mycobacterial culture, first-line susceptibility testing by Bactec MGIT (BD, Franklin Lakes, USA), and real-time PCR assays and second-line susceptibility.

### Study participants and sample collection

Adult patients admitted at KIDH from 2013 to 2014 for both drug-susceptible TB and MDR-TB were eligible for inclusion.

All patients were admitted at KIDH and provided a pre-labeled sputum collection container prior to initiation of anti-TB treatment, and instructed on how to collect an adequate early morning specimen. The specimen quality and volume was inspected by a nurse prior to cold transport to KCRI-Biotechnology. For patients with sputum culture positive for *M.tuberculosis* and initiated on pulmonary MDR-TB treatment, pretreatment demographic and clinical data were extracted from the medical chart. The standardized weight-based daily regimen was prescribed of pyrazinamide, levofloxacin, kanamycin (intramuscular), ethionamide, and cycloserine to complete at least 8 months of inpatient therapy and an additional 12 months of outpatient therapy without kanamycin. Additional susceptibility testing for those agents in the MDR-TB regimen was available from the national reference laboratory, but did not include pyrazinamide. Fluoroquinolone and kanamycin resistance had been rare at KIDH and only one patient had undergone treatment for extensively drug-resistant (XDR)-TB [11]. The majority of patients had sputum collected monthly to determine the time to culture negativity per KIDH protocol. Additionally, the final treatment outcome was determined when available and categorized as treatment failure if death from any cause, treatment interruption, or lack of sputum culture conversion to negative (microbiological failure). Treatment success included treatment completion, and cure (completion plus sustained cultured negativity).

### Sputa processing and PZA susceptibility testing

An equal volume of a mixture of the mucolytic agent N-acetyl-l-cysteine (NALC) and 2% sodium hydroxide (NaOH) was added to the pretreatment sputa. A phosphate buffer was added after 15 min to neutralize the NaOH and prevent the loss of mycobacteria viability. The mixture was centrifuged, the supernatant discarded and the sediment used for culture. Mycobacterial cultures of the pretreatment sputa were performed in the BACTEC mycobacterial growth indicator tubes (MGIT) 960 system. Speciation of *M. tuberculosis* complex was confirmed by MTBc assay [12]. For susceptibility testing, isolates were sub-cultured on to Lowenstein-Jensen (LJ) slants and pure subcultures were added to 4 ml BBL middlebrook 7H9 broth of 4 ml in a sterile tube containing 10 glass beads, vortexed and left to stand for 20 min without disturbing. The supernatant (smooth, free from any clumps) was adjusted to a 0.5 McFarland and 1 ml was diluted by 4 ml of sterile saline (1:5 dilutions). This dilution was used to inoculate the PZA tube by adding 0.5 ml to the PZA containing MGIT vial and incubated in the MGIT 960 machine for up to21 days. Pyrazinamide resistant results were repeated for confirmation [13, 14]. Blood agar plates were simultaneously inoculated to rule out other bacterial contamination, and with each run *M. tuberculosis* H37RV, known to be pyrazinamide susceptible, was included as a control.

Next, the High Resolution Melt (HRM) technique was used to determine the PZA resistance by mutation in the *pncA* gene from the same pure subculture from LJ slants [15]. The HRM assay was performed as previously reported utilizing nine overlapping primer pairs designed to cover the entire *pncA* gene and upstream regions [15, 16]. Each gene segment was individually amplified by real-time PCR followed by HRM analysis. Previously, we have found HRM was 94% concordant to full-length *pncA* sequencing results, and most discrepancies attributable to mixed populations per HRM or transversions [15]. We programmed HRM software to define mutation in *pncA* as < 60% similarity with wildtype sequence.

### Data analysis

For analyses, susceptibility testing by conventional MGIT phenotypic results were compared to *pncA* results and sensitivity, specificity, negative predictive value (NPV) and positive predictive value (PPV) were determined with 95% confidence intervals (CIs). Among MDR-TB patients, pretreatment characteristics and treatment outcomes were compared among those that were susceptible and resistant to PZA. Means were compared using a Student t test, and the standard deviations reported unless otherwise specified, while categorical variables were compared by chi-square tests. The data were analysed using statistical package for social sciences (SPSS) version 23.

## Results

### PZA susceptibility testing by MGIT and *pncA*

Ninety-one *M. tuberculosis* isolates from individual patients were available for analysis. Table 1 summarizes the specificity (94.23%), sensitivity (64.1%), NPV (77.7%) and PPV (89.29%) of *pncA* gene mutation by the HRM method compared to conventional phenotypic testing by MGIT (Table 1). Among discrepancies, 14 had mutation in *pncA* but were susceptible by MGIT PZA. Yet of the 14 susceptible by MGIT PZA but had mutation in *pncA*, 4(28.5%) demonstrated HRM results with percent similarity of 50–59% with the wild type sequence, suggestive of a possible false call of resistance. These possible false calls were found within two assay fragments (*pncA*5 and *pncA*6). Three isolates were PZA resistant by MGIT while *pncA* was wildtype, but no contamination or other phenotypic assay problem was encountered.

**Table 1:** Pyrazinamide susceptibility testing by *pncA* gene mutation compared to conventional phenotypic MGIT methodology (*N* = 91*)

	MGIT PZA Resistant	MGIT PZA Susceptible	Sens (95% CI)	Spec (95% CI)	NPV (95% CI)	PPV (95% CI)
*pncA* mutant	25	14	64.1 (47.18–78.8%)	94.23 (84.05–98.79%)	77.78 (69.59–84.26%)	89.29 (73.05–96.24%)
*pncA*wildtype	3	49				

*MGIT* mycobacterial growth indicator tube, *PZA* pyrazinamide, *NPV* negative predictive value, *CI* confidence interval, *PPV* positive predictive value

*Of these 91, 22 underwent additional Sanger sequencing of *pncA* gene (21 MGIT susceptible and 1 MGIT resistant). 6 carried the silent mutation Ser65Ser, 1 had*pncA* mutation reported previously to be associated with resistance (Leu35Arg), and 14 were wild type including the one isolate with MGIT resistance

The * mark in Table 1 shows the result of 22 isolates that underwent Sanger sequencing.

### PZA resistance among patients with MDR-TB

Among all patients, 30 (32.9%) had MDR-TB (Table 2). Of those with MDR-TB, the mean age was 33 ± 10 years, and 23 (76.7%) were males. Six (20%) were HIV positive, and of those 5 (83%) were on antiretroviral therapy prior to hospitalization for MDR-TB treatment initiation. Twenty five MDR-TB patients (83.3%) had a history of previous TB treatment including patients with > 3 prior episodes. Of the 30 total MDR-TB patients, 15(50%) had isolates with PZA resistance by conventional MGIT testing (Table 2). This proportion expectedly exceeded the number with PZA resistance in the 61 patients without MDR-TB, 13 (21.3%) (*p* = 0.008). Acknowledging the relatively small sample size there were no clinical characteristics that were more common among MDR-TB patients with isolates harboring PZA resistance. When examining the influence on treatment outcome, PZA was the only critical drug in the MDR-TB regimen for which routine susceptibility testing was not available through the national TB treatment program and thus reasonably analyzed independent of other drug-resistance in the regimen for which medications were replaced or dose adjusted. Six (20%) of MDR-TB patients had a poor outcome including treatment failure (Table 3). Among patients with treatment failure, 5 (83%) were PZA resistant compared to only 10 (41.6%) with treatment success (*p* = 0.08). Of those with treatment failure, there were 2 patients that died and both had isolates with PZA resistance. No other pretreatment characteristic was associated with treatment outcome. Detailed clinical information, including prior history of TB treatment was not available for patients treated for non-MDR-TB.

**Table 2:** Clinical characteristics of patients with multidrug-resistant tuberculosis in association with pyrazinamide susceptibility

Characteristics	All *N* = 30	PZA (S) *N* = 15	PZA(R) *N* = 15	*p*-value
Mean age in years ±SD	33 ± 10	34 ± 8	31 ± 12	*P* = 0.44
Gender, Female (%N)	7 (23)	5 (33)	2 (13)	*P* = 0.39
Smoking (%N)	6 (20)	3 (20)	3 (20)	*P* > 0.99
HIV positive (%N)	6 (20)	5 (33)	1 (7)	*P* = 0.17
On ART (%HIV positive)	5 (90)	4 (80)	1 (100)	N/A
Prior TB treatment (%N)	25 (83)	13 (87)	12 (80)	*P* = 0.63
Cavity on chest x-ray (%N)	8 (26.7)	5 (33.3)	3 (20)	*P* = 0.68

*PZA* pyrazinamide, *S* susceptible, *R* resistant, *SD* standard deviation, *HIV* human immunodeficiency virus, *ART* antiretroviral therapy, *TB* tuberculosis

**Table 3:** Characteristics associated with MDR-TB treatment outcomes

Clinical/microbiological characteristic	Treatment success *N* = 24	Treatment failure *N* = 6	*p*-value
Mean age years ±SD	34 ± 10	28 ± 9	*P* = 0.20
Gender, Female (%N)	6 (25)	1 (16.7)	*P* = 0.57
HIV infected (%N)	5 (20.8)	1 (20)	*P* = 0.66
Smoking (%N)	6 (25)	0	*P* = 0.33
Prior treatment	21 (87.5)	4 (66.7)	*P* = 0.25
Cavity on chest x-ray	6 (25)	2 (33.3)	*P* = 0.65
PZA resistant (%N) by MGIT PZA	10 (41.6)	5 (83)	*P* = 0.08

*SD* standard deviation, *HIV* human immunodeficiency virus, *PZA* pyrazinamide

## Discussion

In this cohort of consecutively enrolled patients at a referral hospital in Tanzania, PZA resistance was common and occurred in approximately one in five patients treated for drug-susceptible TB and half of all patients treated for MDR-TB. Among MDR-TB patients prone to worse treatment outcomes, PZA resistance was commonly observed among those with treatment failure. Therefore, PZA susceptibility testing appears to be a critical adjunct to TB care in particular for determining whether or not a patient should be initiated on a regimen utilizing novel drugs or combinations to shorten the total treatment duration [17]. This finding is relevant given that in Tanzania, as in other TB endemic settings, MDR-TB treatment availability has not been introduced with a complementary increase in laboratory capacity for drug susceptibility testing for all drugs in a potential regimen.

In this study we compared two methods of PZA susceptibility testing, and found the HRM assay for *pncA* gene mutation to have good PPV and adequate NPV for predicting PZA resistance against the conventional phenotypic method. Prior studies have demonstrated that *pncA* mutation does not entirely account for all phenotypic PZA resistance, whereby sequencing of *pncA* yields about 81–83% sensitivity and 81–90% specificity in two of the largest studies [18, 19]. Given the importance of identifying the exact *pncA* mutation, since some are very high confidence mutations, while others high, undetermined, or not associated with resistance, future work of ours involves exploring TaqMan probe based assays instead of HRM [20]. Conventional susceptibility testing in MGIT is cumbersome given the need for specialized acidic media for PZA and is further limited by cost and prolonged turnaround time. So while there are theoretic advantages to a more rapid molecular method such as HRM that can be performed in a laboratory with a real-time PCR cycler, this study highlights the potential limitation of molecular assays that do not provide sequence specific information.

While reports of PZA resistance that near 50% in MDR-TB are frequent, the proportion of patients in our study without MDR-TB that had PZA resistance (21%) was unusually higher than seen in most settings [21], for example PZA resistance was found in 10.5% of isolates from randomly sampled clinics in Nairobi, Kenya, and only 2.9% of new cases and 11.1% of retreatment cases from 79 TB patients in the Tanga region of Tanzania. Therefore, our study represents the largest of its kind to date in Tanzania, and raises concern for the potential inactivity of PZA at a much higher proportion in the Kilimanjaro region of the country, a referral area for both MDR and non-MDR-TB. Of further support that these patient’s isolates are truly resistant, 25 (89.2%) of conventionally PZA resistant isolates also had *pncA* mutation by the HRM method. One possibility is that several of these were *M.bovis*; however this seems unlikely because we have not noted a high prevalence previously, and many of the HRM results showed *pncA* variance that derived from regions elsewhere from amplicon 3 where the His57Asp *pncA* mutation of *M. bovis* resides. Thus given this high rate of true PZA resistance, we plan to examine if this is a more local epidemiological phenomenon. For instance, KIDH, the referral hospital for this study, serves a large population working in small scale mines in concentrated geographic area for which multiple episodes of TB and the need for retreatment are common and could be explained by a higher rate of relapse if those patients are treated with PZA without the clinician knowing of PZA resistance [11]. The high rate of PZA resistance is clearly clinically relevant, since knowledge of PZA resistance would prompt an extension of treatment to 9 months for otherwise drug-susceptible TB, but this is rarely done in Tanzania and other settings where susceptibility testing for PZA is not routine and monitoring of treatment response relies on sputum smear microscopy [22].

The study is limited in that not all *M. tuberculosis* isolates underwent Sanger sequencing of the *pncA* gene to interrogate the MGIT and HRM discrepancies due to funding constraints. Pyrazinamidase activity assays that have been used to resolve similar discrepancies in prior studies were not able to be performed [23]. Furthermore, susceptibility testing for other drugs in the regimen was not part of the original protocol and so results could not be compared against those the clinician may have used to adjust a patient’s drug regimen and this may be particularly important for components of the regimen such as the fluoroquinolones. Yet given that PZA was the one drug that was continued empirically for MDR-TB treatment, we believe it was valid to include this categorical variable as a potential predictor of treatment failure. Overall, this was a sample size of convenience and not powered to detect differences that may have been observed with a larger cohort.

## Conclusion

Susceptibility testing for PZA at this referral hospital in Tanzania could drive decisions on the most efficacious use of novel drug regimens for MDR-TB, and the surprisingly high proportion of PZA resistance among cases without MDR-TB requires more urgent local investigation.

## Recommendation

As PZA susceptibility testing appears to be a critical adjunct to TB care, more comprehensive studies are needed to determine if conventional phenotypic testing on the MGIT platform can effectively be replaced with sequencing of the *pncA* gene, or other molecular alternatives.

## Abbreviations

DST: Drug susceptibility testing
HRM: High Resolution Melting Analysis
KCMUCo: Kilimanjaro Christian Medical University College
KCRI: Kilimanjaro Clinical Research Institute
KNTH: Kibong’oto National Tuberculosis hospital
MDR-TB: Multidrug-resistant Tuberculosis
MGIT: Mycobacteria Growth Indicator Tube
PZA: Pyrazinamide
TB: Tuberculosis
XDR-TB: Extensively drug-resistant tuberculosis
